# Fatigue Behavior of Multi/Unit-Supported Dental Restorations: Implant Platform vs. Prosthetic Platform

**DOI:** 10.3390/dj13080374

**Published:** 2025-08-18

**Authors:** Eduardo Anitua, Mikel Armentia, Ernest Mallat, Beatriz Anitua

**Affiliations:** 1 Eduardo Anitua Foundation, 01007 Vitoria, Spain; eduardo@fundacioneduardoanitua.org; 2 R&D Department, Biotechnology Institute I Mas D S.L, 01005 Vitoria, Spain; 3 SCOE Foundation, 08001 Barcelona, Spain; emallat@clinicamallat.com; 4 University Institute for Regenerative Medicine and Oral Implantology (UIRMI), University of the Basque Country (UPV-EHU), 01006 Vitoria, Spain; bzanitua@gmail.com

**Keywords:** dental implants, prosthetic components, Finite Element Analysis (FEA), mechanical behavior, multi/unit abutment

## Abstract

The increasing popularity of Multi/Unit abutments in dental restorations is attributed to their clinical advantages, yet little is known about their mechanical behavior, particularly in terms of fatigue performance. **Background/Objectives:** This study aimed to evaluate the mechanical behavior of Multi/Unit abutments with a focus on the impact of implant and prosthetic platform diameters on fatigue performance. **Methods:** Five dental restoration models were analyzed using Finite Element Analysis by incorporating implants of identical length and body diameter but varying implant platform size (3.5 and 4.1 mm) and prosthetic platform size (3.5, 4.1, and 5.5 mm). Mechanical stresses on critical sections of the screws were assessed under cyclic loads. **Results:** The results revealed that the implant platform diameter had minimal influence on the fatigue performance of the prosthetic screw, while a wider prosthetic platform significantly improved its mechanical behavior by reducing stress and allowing the use of larger screw metrics. These findings emphasize that the prosthetic platform diameter plays a crucial role in protecting the prosthetic screw, which is often the critical component in dental restorations that use Multi/Unit abutments. **Conclusions:** The study highlights the importance of carefully selecting platform dimensions to optimize the mechanical performance and longevity of dental restorations utilizing Multi/Unit abutments.

## 1. Introduction

Implant-supported prostheses, either screw- or cement-retained, are a widely used option to replace single teeth as well as several teeth [1]. They have traditionally been composed of an implant, an abutment, and a screw that joins both parts, providing structural integrity to the restoration [2,3,4]. This type of dental restoration is known as implant-supported, directly attached, or direct-to-implant restoration.

Nevertheless, in direct-to-implant restorations, the implant connection is covered and uncovered several times during impression taking, the fitting of the restoration, its placement for hygienization purposes, and so on [5]. This may result in a negative bone response that may lead to bone loss at the marginal ridge level accompanied by apical soft tissue migration [6].

To avoid this problem, the “one-abutment one-time” protocol was presented. This protocol proposes the permanent placement of the final abutment as soon as the implant is inserted into the bone of the patient, thus eliminating the multiple disconnections and reconnections of the implant–abutment connection (IAC). As a result, the seal and stability of the bone and soft tissue surrounding the IAC is not disrupted, avoiding or reducing, in this way, bone and soft tissue loss [5,6,7].

For this reason, the use of Multi/Unit (also known as transepithelial) abutments is an increasingly common practice that brings numerous advantages from a clinical point of view. First, it represents the perfect option for the “one-abutment one-time” protocol because the Multi/Unit abutment is placed immediately after the implant insertion, ensuring that sealing is uninterrupted [8]. Moreover, it allows variances in the prosthetic emergence and the abutment height, given that it is able to adapt to each prosthetic case, even once the implant has been inserted [9].

Clinical studies report that most fatigue failures in conventional implant-supported restorations occur in screws [10,11], leading to a loss of structural integrity and, ultimately, the premature failure of the restoration [3,4]. Thus, introducing a Multi/Unit abutment also offers a great biomechanical advantage, as it often causes the mechanical fuse (the component that would fail in case of overload or unexpected failure) to be the prosthetic screw that is mounted on the Multi/Unit abutment instead of the screw that goes directly into the implant. Moving the failure away from the bone level and protecting the implant provides a higher level of safety and success [12].

Despite the advantages and the increasing use of Multi/Unit abutments, few biomechanical studies of the aforementioned components were found. There are, however, studies that analyze the mechanical behavior of direct-to-implant restorations. These studies examine how different geometric variables—i.e., implant length, implant diameter, platform diameter, type of IAC, external thread profile, etc.—affect the mechanical behavior. As a general conclusion, it can be stated that length is not particularly relevant in terms of stresses or strains in the bone or from the point of view of implant strength, or at least it is much less relevant than the rest of the parameters [13,14,15,16]. Increasing the diameter of the implant body, which is obviously limited by the interdental and bone space, has been shown to be decisive in reducing the stresses transmitted to the bone [17,18,19], those suffered by the resistant section of the implant [14,20,21], and those suffered by the prosthetic screw [22]. However, the geometry of the external threads also has a considerable influence on stress distribution in the surrounding bone. Thread depth, pitch, and flank angle determine how occlusal forces are transmitted to the cortical and trabecular bone, directly affecting both primary stability and the risk of crestal bone loss. For instance, deeper threads and those with a lower pitch tend to generate more favorable stress patterns under axial and oblique loads [23,24]. Increasing the diameter of the implant platform is also a parameter that positively affects the mechanical behavior of the dental restoration by enhancing implant–abutment joint strength and reducing stresses in the retaining screw [22,25,26]. However, reducing this diameter by applying the platform switching concept can help to preserve the peri-implant bone [25]. Therefore, a compromise between the two approaches should be reached in order to arrive at the optimal solution. Regarding the type of connection, although it is not possible to generalize because it will depend on how the connection is designed and manufactured, studies suggest that internal connection provides a better seal against bacterial filtration [27], better aesthetics, and the possibility of applying platform switching options [28]. In addition, several studies have found that internal connection provides better mechanical performance than external connection [29,30,31].

However, no literature was found studying how variables such as prosthetic platform diameter or implant platform diameter affect the mechanical behavior of the dental restoration when mounting a Multi/Unit abutment. To satisfy this need for knowledge about the mechanical behavior of these types of restorations, this study precisely analyzes via Finite Element Method (FEM) the effect of varying both implant and prosthetic platform sizes on the fatigue behavior of Multi/Unit-supported restorations.

## 2. Materials and Methods

**Figure 1:**
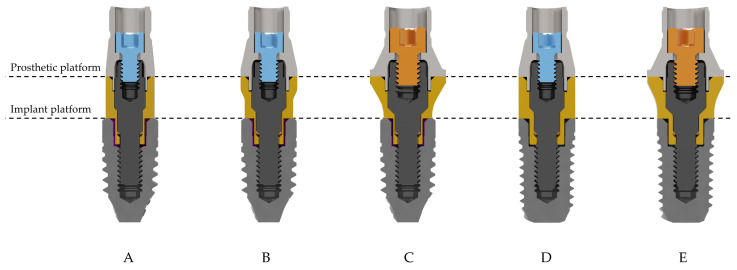
Dental restorations under study. (**A**), IIPECA4075 + INTMIUPE3530 + CPMIUPE. (**B**), IIPECA4075 + INTMIUPE30 + CPMIUPU. (**C**), IIPECA4075 + INTMIUPEEX30 + CPMIUPA. (**D**), IIPUCA4075 + INTMIUPU30 + CPMIUPU. (**E**), IIPUCA4075 + INTMIUPUEX30 + CPMIUPA.

Five different dental restorations have been evaluated (BTI Biotechnology Institute S.L., Vitoria, Spain). These restorations were composed of only two implant models of the same length (7.5 mm), the same implant body diameter (4 mm), and different implant platform diameters (3.5 and 4.1 mm). Multi/Unit abutments with different prosthetic platform diameters (3.5, 4.1, and 5.5 mm) were mounted on these implants. Prosthetic cylinders were mounted over the Multi/Unit abutments and retained by means of two different prosthetic screws—M1.4 screws for the 3.5 mm and 4.1 mm platforms, and M1.8 screws for the 5.5 mm platforms. Figure 1 shows the 5 dental restorations with each of the prosthetic components used, detailed in Table 1. All implants, cylinders, and Multi/Unit abutment bodies were manufactured from commercially pure grade 4 titanium (Ti CP4), whereas all the screws—including those supplied with the Multi/Unit abutments—were made of Ti6Al4V extra-low interstitials (ELI) alloy (Ti grade 5), with the chemical composition detailed in Table 2.

**Figure 2:**
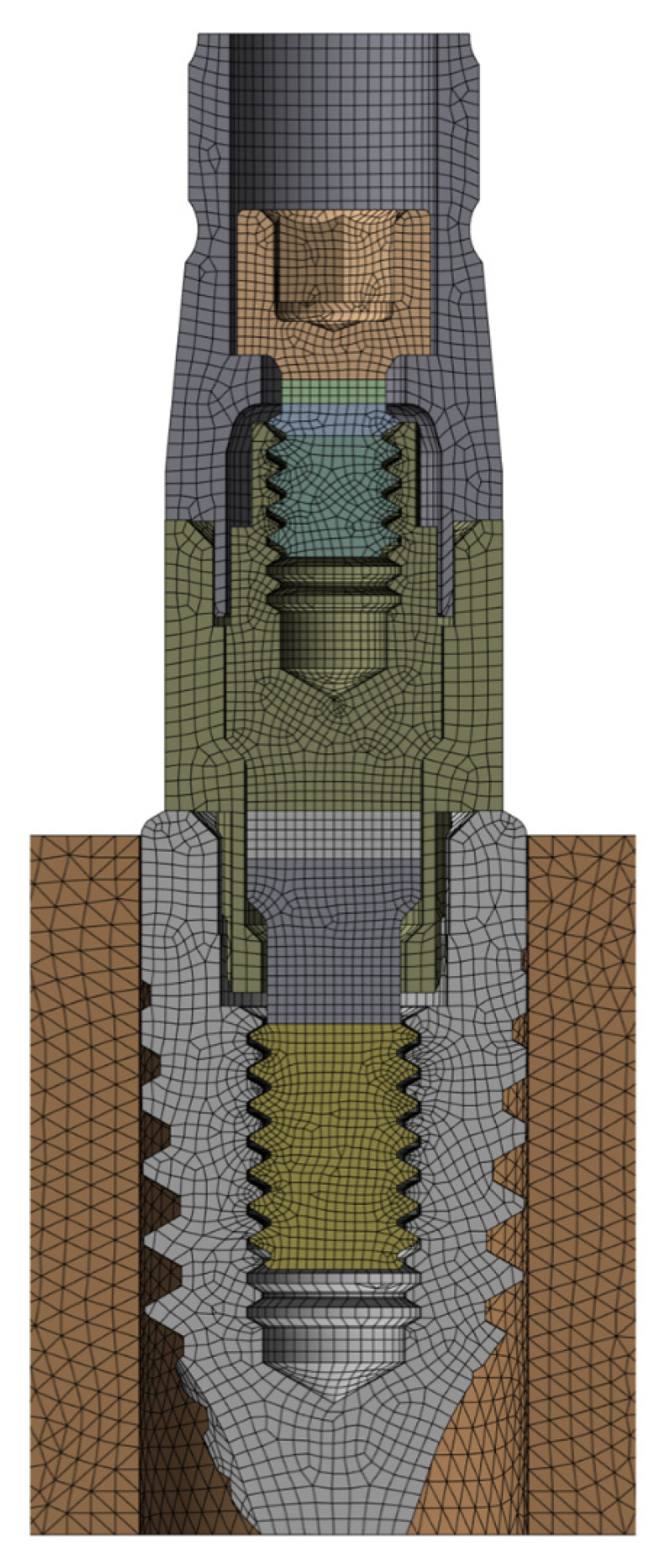
FEA meshed model of A dental restoration.

Figure 2 shows the meshed model of one of the five dental restorations analyzed in the study, with approximately 1.2 M Degrees of Freedom (DoF). Finite Element Analyses (FEAs) were performed in Ansys Workbench^®^ 19R1 (Ansys Iberia S.L., Madrid, Spain). A (cyclic) chewing force was simulated with an inclination of 30° with respect to the vertical, as indicated in ISO 14801 [32]. In order to consider a more compromised, and possibly also more realistic, case, the load was applied at 10.5 mm height from the IAC. Taking advantage of the symmetry of both the geometry and the applied load, only half of the assembly was modelled, and consequently, half of the preload and external load was applied. The screw threads and the internal thread of the implants were modelled as cylindrical rather than helical, as this simplification introduces a maximum error of less than 3.5% [33]. Both materials were modelled as linear elastic. For commercially pure grade 4 titanium (CP4), an elastic modulus of E = 103 GPa and Poisson’s ratio ν = 0.35 were used; for Ti6Al4V ELI (grade 5), ν = 0.31 was applied. Contacts were defined as frictional contacts, with a coefficient of friction of 0.17 for screw–implant and screw–Multi/Unit abutment contacts, and 0.21 for implant–Multi/Unit abutment and Multi/Unit abutment–cylinder contacts [34].

**Table 2:** Chemical composition of materials used in implants and prosthetic components according to [35,36].

Ti6Al4V ELI (Ti Gr5)		Ti CP4	
Composition	Wt. %	Composition	Wt. %
Al	5.5–6.5	N(max)	0.05
V	3.5–4.5	C(max)	0.08
Fe(max)	0.25	Fe(max)	0.5
O(max)	0.13	O(max)	0.4
C(max)	0.08	H(max)	0.0125
N(max)	0.05	-	-
H(max)	0.012	-	-

The FEAs performed consisted of three load steps (see scheme (1)). In the first load step, the preload is applied to the screw of the Multi/Unit abutment, i.e., the lower one that is connected to the implant and seals the connection between the Multi/Unit abutment and the implant. In the second load step, the preload of the prosthetic screw that is screwed on the Multi/Unit abutment, i.e., the one that connects the cylinder and the Multi/Unit abutment, is introduced. Both preloads are introduced into the model by defining a pretension section and imposing an adjustment equivalent to an axial preload. This preload is calculated using the Motosh formula [33], resulting in 688 N for the screw connecting the Multi/Unit abutment and implant tightened at 35 Ncm, 572 N for the M1.4 prosthetic screw (TTMIR) tightened at 20 Ncm, and 698 N for the M1.8 prosthetic screw (TTMIUPA) tightened at 30 Ncm. In the third load step, the masticatory load is applied using an increasing slope from 0 to 400 N.

(1) $$\mathrm{FEA}\;\mathrm{steps}\left\{\begin{array}{c} 1^{\mathrm{st}}\;\;\;\;\to \;\;\;Preload\;of\;the\;lower\;screw\;\;\;\;\\ 2^{\mathrm{nd}}\;\;\;\to \;\;\;Preload\;of\;the\;upper\;screw\;\;\;\;\\ 3^{\mathrm{rd}}\;\;\;\;\to \;\;\;Masticatory\;load\;application\; \end{array}\right.$$

**Figure 3:**
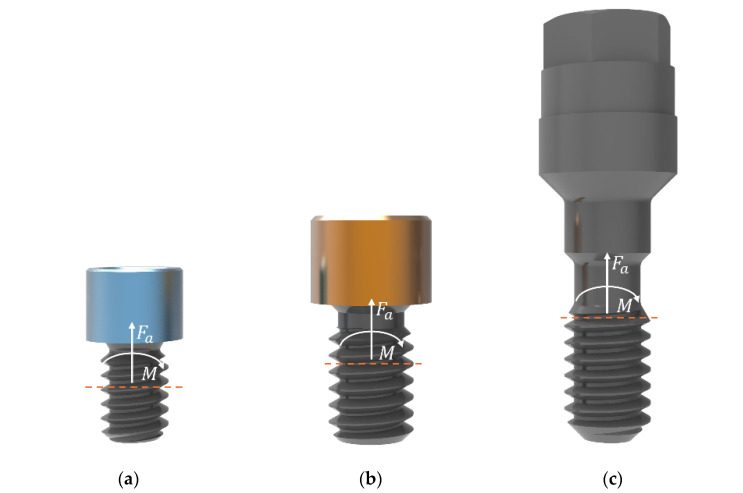
Critical section (orange dashed lines) and force reactions (axial force and bending moment) of each screw type: (**a**) TTMIR, (**b**) TTMIUPA, and (**c**) Multi/Unit screw.

From the FEA, the force reactions—axial force and bending moment—were obtained for the critical section of the screw, that is, for the first thread in contact (see Figure 3), in order to then calculate the nominal stress in that section by using the following expression from the Theory of Elasticity for uniaxial stress:

(2) $$\sigma_{nom}(F)=\frac{F_{a}}{A}+\frac{M\cdot r}{I}$$

where $\sigma_{nom}$ is the nominal stress obtained for a certain masticatory load F, $F_{a}$ is the axial force, M is the bending moment, A is the net section, I is the inertia of the net section, and r is the radius of the net section. Nominal stress values for each masticatory load value and its corresponding 10% of that value were calculated. Then, mean $\sigma_{m}$ and alternating $\sigma_{a}$ components of the nominal stress were obtained as follows:

(3) $$\begin{array}{c} \sigma_{m}=\frac{1}{2}(\sigma_{max}+\sigma_{min})\\ \sigma_{a}=\frac{1}{2}(\sigma_{max}-\sigma_{min}) \end{array}$$

where $\sigma_{max}$ is the maximum stress and $\sigma_{min}$ is the minimum stress. This was obtained for the entire 0–400 N range of masticatory load mentioned previously. For the evaluation of the fatigue behavior of the prosthetic screw, the Walker criterion [37] was used because it has been proven to fit experimental results well once fitted according to the procedure described in Dowling et al. [38] in a previous work [33]. The Walker effective stress for fatigue calculations were carried out as follows:

(4) $$\sigma_{a_{eq}}=\sigma_{a}\cdot {\left(\frac{2}{1-\frac{\sigma_{min}}{\sigma_{max}}}\right)}^{1-\gamma }$$

where γ is 0.21, as calculated in a previous work [33], and $\sigma_{a_{eq}}$ is the Walker alternating equivalent stress for fatigue calculations.

## 3. Results

**Figure 4:**
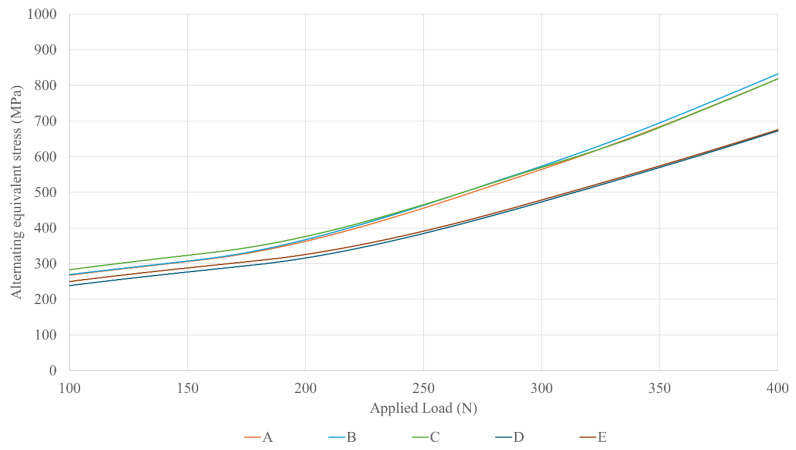
Effective stress of the lower screw versus the external applied load across different dental restorations (A–E).

Figure 4 shows the effective fatigue stress of the critical section of the lower screw for a masticatory load range from 100 to 400 N. The lower screws of restorations A, B, and C, which have Ø3.5 mm implant platforms and different prosthetic platforms, show practically the same stress level at the critical section. Moreover, the lower screws of restorations D and E, which have Ø4.1 mm implant platforms and different prosthetic platforms, also suffer same stress status at the critical section. In general, the lower screw undergoes a higher level of stress as the implant platform narrows.

**Figure 5:**
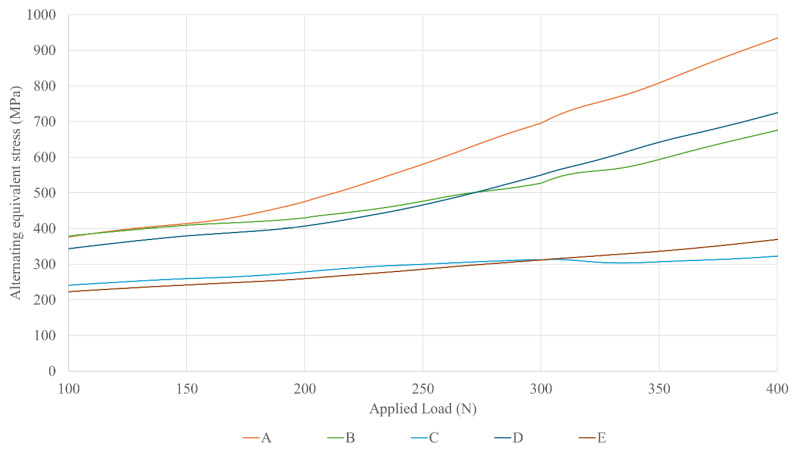
Effective stress of the upper screws versus the external applied load across different dental restorations (A–E).

Figure 5 shows the effective fatigue stress of the critical section of the prosthetic screw mounted on the Multi/Unit abutment for the same load range, from 100 to 400 N. The upper screws of restorations B and D, which have Ø4.1 mm prosthetic platforms and different implant platforms, show similar levels of stress at the critical section. The same occurs with the upper screws of restorations C and E, which have Ø5.5 prosthetic platforms and different implant platforms and exhibit a similar stress status. In general, the upper screw undergoes a higher level of stress as the prosthetic platform narrows.

## 4. Discussion

Concerning the lower screw, the prosthetic platform diameter has practically no influence in its mechanical behavior, whereas implant platform diameter does have an influence, although it may be considered small for loads below approximately 175 N. Moreover, it was determined in a previous work that the fuse of dental restorations using a Multi/Unit abutment is usually located at the upper screw [12]. For this reason, the influence of the implant platform on the lower screw should not be decisive, as it is not expected to be the fuse/failing component of the restoration.

Nevertheless, R&D departments should carefully design their restorations to ensure that the fuse is properly located. This may be accomplished in several ways: by ensuring that at the critical section, the lower screw suffers lower stresses than the upper screw; by selecting a material [39,40] or coating [41] for the lower screw that provides better mechanical properties; or by using a manufacturing process that gives better mechanical properties to the critical section of the lower screw, such as rolled threaded prosthetic screws that provide better mechanical performance than cut threaded screws [42].

Regarding the upper screw—the one expected to be most relevant according to the previous discussion—the implant platform diameter has very little influence on its mechanical behavior. In contrast, the prosthetic platform plays a significant role: a wider platform provides better protection against external loads. This effect presents two distinct trends. Firstly, there is an initial difference between the expanded Ø5.5 mm prosthetic platform and the other configurations. This can be attributed to the use of a larger prosthetic screw (M1.8 instead of M1.4), which naturally leads to lower stress values and the improved mechanical performance of the restoration. Secondly, another trend emerges between 175 and 200 N, where the slope of the curves change, and the lines begin to diverge. This represents the mechanical shielding effect of the prosthetic platform, with wider platforms offering greater protection to the screw. As shown in the results, this effect becomes especially pronounced at masticatory loads above 250 N, at which point stress differences between the configurations increase substantially: a 19% reduction in stress occurs when switching from a Ø3.5 mm to a Ø4.1 mm prosthetic platform, and a 50% reduction when occurs when switching from Ø3.5 mm to Ø5.5 mm.

These results are in line with the conclusions obtained in previous works, even though they only analyzed directly attached restorations. Shadid et al. concluded that mechanical behavior is negatively affected by the reduction of the contact diameter of the IAC [26]. Minatel et al. [25] demonstrated that reducing the diameter of the implant–abutment connection (IAC) leads to increased stress concentration in the retaining screw. Similarly, Nicolás-Silvente et al. conducted experimental fatigue tests in which screw failure was the most common mode of failure. Their results showed that wider platforms yielded higher fatigue limits, although this conclusion may be partially limited by the fact that the tested specimens featured different connection geometries [43]. Armentia et al. performed FEAs and experimental fatigue tests on different dental restorations, concluding that an increase in implant body diameter, and especially implant platform diameter, enhances the fatigue life of the prosthetic screw and, consequently, the whole dental restoration [22].

It is worth mentioning that each screw is mainly influenced by the platform closest to it. Specifically, the lower screw is affected primarily by the implant platform, while the prosthetic screw is influenced mainly by the prosthetic platform. Assuming the dental restoration is designed so that the prosthetic screw acts as the fuse of the system, the effect of the implant platform can be considered negligible. In contrast, the prosthetic platform diameter plays a key role. Therefore, when clinically appropriate, it is advisable to opt for wider prosthetic platforms to protect the fuse of the system—the prosthetic screw—and thus increase the overall lifespan of the restoration.

Additionally, expanded prosthetic platforms not only provide better protection for the prosthetic screw, resulting in more durable restorations, but also allow the use of prosthetic screws with a larger thread diameter (metric), further enhancing their mechanical performance.

As a result, the screw experiences less stress due to the combination of two key factors. First, the wider platform offers improved shielding from external loads. Second, the screw itself, having a larger diameter, is inherently more resistant to mechanical failure. The synergy of these two factors significantly extends the lifespan of dental restorations supported by expanded prosthetic platforms. Future research should explore additional variables such as Multi/Unit abutment heights, occlusal load distributions, and different implant–abutment connection designs to further validate and expand upon these results.

## 5. Conclusions

This study demonstrates that each screw in a Multi/Unit-supported dental restoration is primarily influenced by the platform closest to it: the implant platform affects the lower screw, while the prosthetic platform influences the prosthetic screw (upper screw). Considering that the prosthetic screw often acts as the mechanical fuse of the restoration, the implant platform diameter has a limited effect on fatigue behavior. By contrast, the prosthetic platform diameter plays a critical role. Wider prosthetic platforms not only provide better protection against masticatory loads but also enable the use of prosthetic screws with larger screw metrics, further improving their mechanical resistance. The combination of these factors significantly increases the overall longevity of the restoration. These findings underscore the importance of carefully selecting prosthetic platform dimensions in clinical practice to optimize the durability and mechanical reliability of Multi/Unit-supported restorations. Based on these conclusions, the following recommendations can be made to improve the mechanical performance of implant-supported restorations:

1. Use Multi/Unit abutments to shift the mechanical fuse away from the bone level.
2. The implant platform diameter has little mechanical influence on screw fatigue.
3. The prosthetic platform diameter has a strong mechanical impact: expanded prosthetic emergence profiles are recommended to improve the mechanical performance of the restoration.

## Figures and Tables

**Table 1:** Components and main parameters of the restorations under study.

	A	B	C	D	E
Implant	IIPECA4075	IIPECA4075	IIPECA4075	IIPUCA4075	IIPUCA4075
Body Ø (mm)	4.0	4.0	4.0	4.0	4.0
Implant platform Ø (mm)	3.5	3.5	3.5	4.1	4.1
Multi/Unit abutment	INTMIUPE3530	INTMIUPE30	INTMIUPEEX30	INTMIUPU30	INTMIUPUEX30
Tightening torque (Ncm)	35	35	35	35	35
Prosthetic platform Ø (mm) *	3.5	4.1	5.5	4.1	5.5
Cylinder	CPMIUPE	CPMIUPU	CPMIUPA	CPMIUPU	CPMIUPA
Prosthetic screw	TTMIR	TTMIR	TTMIUPA	TTMIR	TTMIUPA
Screw metric	M1.4	M1.4	M1.8	M1.4	M1.8
Tightening torque (Ncm)	20	20	30	20	30

* The 3.5 mm prosthetic platform was not considered for IIPUCA4075, as there are no such solutions available on the market and it is not prosthetically viable.

## Data Availability

Data is unavailable due to privacy restrictions.

## Abbreviations

The following abbreviations are used in this manuscript:

IAC	Implant-Abutment Connection
FEM	Finite Element Method
FEA	Finite Element Analysis
DoF	Degrees of Freedom
